# Influence of selective phrasing on consent to DNAR

**DOI:** 10.1186/s12910-026-01548-7

**Published:** 2026-07-14

**Authors:** Kiichi Inarimori, Yugo Maeda

**Affiliations:** 1 Hiroshima University, Higashi-Hiroshima, Japan; 2 Nagoya University of Commerce & Business, Nisshin, Japan

**Keywords:** Nudge, Libertarian Paternalism, Experimental Bioethics, DNAR, Informed Consent

## Abstract

**Background:**

Within the framework of libertarian paternalism, nudges have been proposed as a way to guide decision-making while preserving individual autonomy, often by exploiting cognitive biases such as framing effects. Although nudges are commonly discussed as a promising means of promoting patients’ best interests, their practical application in real medical contexts remains limited. This study examines whether physicians’ selective phrasing, as a more direct form of intervention than conventional nudging, influences surrogates’ decisions regarding “do not attempt resuscitation” (DNAR) orders.

**Methods:**

We conducted a series of vignette-based online experiments in which participants evaluated hypothetical end-of-life scenarios. We tested whether subtle linguistic variations in physicians’ explanations—such as mentioning potential harms of cardiopulmonary resuscitation (CPR) or associating DNAR with “naturalness”—affected participants’ consent to DNAR.

**Results:**

Across studies, participants’ willingness to consent to DNAR was significantly influenced by selective phrasing. Specifically, briefly highlighting the potential harms of CPR and framing DNAR as a form of “natural end-of-life care” were associated with higher rates of DNAR acceptance.

**Conclusions:**

The findings demonstrate that physicians’ language plays a crucial role in shaping end-of-life decisions without imposing explicit constraints on choice. This suggests that selective phrasing may function as a form of nudging in the broader sense, raising important ethical questions about the boundaries of permissible linguistic selectivity in medical communication.

**Supplementary Information:**

The online version contains supplementary material available at https://doi.org/10.1186/s12910-026-01548-7.

## Background

This section aims to define and situate selective phrasing, Which is the main focus of this paper, within the broader debate on nudging in medicine.

Within the framework of libertarian paternalism, the “nudge” approach is often regarded as a promising way to balance patient autonomy with their best interests in medical decision-making [5, 18]. A nudge is typically characterized as a form of intervention in “choice architecture,” which steers people’s decisions through situational factors without limiting their options [18]. However, critics argue that the use of nudges compromises patients’ autonomy [16], undermines rationality [4, 9], or violates the disclosure requirements of informed consent because it is incompatible with truth-telling [17, 20]. In contrast, some scholars contend that consent influenced by cognitive biases is no less valid than everyday decisions shaped by similar factors [6]. This has led to ongoing questions about the conditions under which nudging might be ethically justifiable [10, 14]. Whether nudging in general undermines patients’ autonomy in medical contexts remains an open question.

Beyond these theoretical debates concerning the moral permissibility of nudging in medical contexts, a separate issue concerns the practical feasibility of nudges in the narrow sense. We define nudges in the narrow sense as intentional alterations of choice architecture designed to exploit cognitive biases or heuristics. The use of probabilistic framing (e.g., presenting a treatment as having a 90% success rate rather than a 10% failure rate in order to increase patients’ willingness to accept it) is a representative example of nudging in the narrow sense. Since people tend to be loss-averse, presenting the success rate of a medical treatment is likely to increase patients’ acceptance of that treatment [19].

However, the precise probabilities associated with a particular treatment are often difficult to determine, as outcomes depend heavily on a patient’s unique clinical trajectory and changing clinical circumstances. Some forms of narrow nudging (e.g., probabilistic framing) face difficulties in clinical settings due to such substantial epistemic uncertainty.

To illustrate this point, consider end-of-life care. Medical practitioners often recommend that a DNAR (do-not-attempt-to-resuscitate) order be put in place to avoid prolonged suffering and medically futile interventions. DNAR decision-making is a prominent example of informed consent in contemporary medicine. Nevertheless, the feasibility of employing probabilistic framing in this context is often limited. The success rate of CPR is strongly influenced by factors such as staffing availability and the time required to initiate resuscitation, while outcomes following successful CPR depend heavily on patients’ age, underlying medical conditions, and prior medical history. These considerations may make probabilistic framing difficult to employ in real-world clinical settings, even when clinicians reasonably judge that resuscitation would not benefit the patient overall. Although clinicians may use more qualitative probabilistic expressions (e.g., “CPR is highly likely to fail”), they may hesitate to employ strongly probabilistic language when the available evidence does not clearly justify highly definitive claims.

Given the importance of DNAR decision-making in contemporary medicine, these limitations of explicit probabilistic framing motivate consideration of broader forms of nudging in clinical contexts (i.e. nudges that do not solely rely on cognitive biases). What counts as a nudge in medicine is contested, and some scholars argue for a wider conception that goes *beyond* the exploitation of cognitive biases. For instance, Douglas and Proudfoot [7] argue that physicians’ recommendations constitute a form of nudging because they significantly shape patients’ choices without coercion. Similarly, Muñoz and colleagues [13] contend that the informed consent process itself can serve as a kind of nudge, as it steers and directs patients toward what physicians believe is the best option.

Whether these interventions in patients’ decisions, going beyond the use of cognitive biases and heuristics, should count as *nudges* is controversial, and settling this issue is beyond the scope of this paper. Nevertheless, for the sake of argument, we presuppose a meaningful distinction between nudging in the narrow sense, which involves interventions in choice architecture through cognitive biases or heuristics, and nudging in the broader sense, which allows for more direct forms of intervention.

Here, we define nudging in the broader sense within medical contexts as any non-coercive intervention that intentionally influences the decision-making process of patients or their surrogates by structuring how options are presented, interpreted, or evaluated. Such influence may incorporate, but is not limited to, the utilization of cognitive biases. Under this broader conception, nudging encompasses explicit professional recommendations, the selective phrasing of medical information, communicative emotional cues, and other forms of intentional, non-coercive influence that steer decision-making without eliminating choices or imposing substantial penalties.

Some clarifications are in order.

First, our proposal should not be understood as a rejection of narrow nudges based on cognitive biases. We do not contend that nudging in the narrow sense (i.e., the utilization of cognitive biases) is ineffective. Rather, our position is that nudging in the broader sense can supplement and strengthen libertarian paternalistic interventions within clinical decision-making processes.

Second, we do not presuppose that nudging in the narrow sense and the broad sense are mutually exclusive. Rather, it is reasonable to expect that nudging in the broader sense can cause cognitive biases in some cases. For instance, when a doctor emphasizes the beneficial aspects of a treatment using a specific phrase, whether she states it at the beginning of the explanation or at the end of it may cause order effects. The critical point is that nudging in the broader sense includes libertarian interventions into the decision-making process that go beyond the mere exploitation of cognitive biases alone.

Third, nudging in the broad sense should be distinguished from explicitly autonomy-violating manipulation or coercion, which involves influence through “irresistible threats” and is morally impermissible in most cases [2]. Just as narrow nudges steer patients’ choices non-coercively, broad nudges also steer patients’ choices while leaving sufficient room for them to act contrary to physicians’ recommendations. Similarly, broad nudges should be distinguished from attempts to manipulate patients’ decision-making through fraudulent disclosure. Morally permissible forms of broad nudging should satisfy standard disclosure requirements and support, rather than impair, patients' capacity for informed decision-making.

The focus of this paper is on one such broader form of nudging: physicians’ selective phrasing. In certain medical contexts, such as end-of-life care, it may be inappropriate or difficult for physicians to explicitly recommend a particular option, especially when it involves the withdrawal of treatment, such as a DNAR order. Recommendations for treatment withdrawal are therefore often conveyed implicitly. One prominent form of such implicit recommendation is “selective phrasing,” which we define as the intentional formulation of words and expressions used to present information during the disclosure process. For example, a physician might emphasize the suffering caused by resuscitation (“CPR may cause her to suffer even more”) to encourage a DNAR order, refraining from mentioning potential benefits (“CPR may slightly prolong your mother’s life”).

The original contribution of this paper is to demonstrate the influence of physicians’ selective phrasing in end-of-life care and to examine the potential of nudging in the broader sense. Previous studies, such as Malloy et al. [11], have already shown that the way physicians describe CPR affects people’s choices in advance directives; however, their focus was limited to consent for specific treatments, such as mechanical ventilation, rather than consent to DNAR itself. Moreover, their research primarily examined substantial differences in informational content, whereas this study focuses on the effects of subtle linguistic variations. Using online experiments with hypothetical scenarios, we investigated how selective phrasing influences consent to DNAR—a paradigmatic case of informed consent in which patient autonomy is especially important. Our findings suggest that physicians’ intentional formulation of words and expressions can significantly influence end-of-life decisions, raising the ethical question of how selective medical practitioners should be in their explanations.

## Methods

### Overall design

We conducted three vignette-based online experiments to examine how selective phrasing influences individuals’ consent to DNAR. Across all studies, the primary outcome was participants’ agreement with DNAR (binary: agree/do not agree). All studies included comprehension checks to ensure scenario understanding. To assess the association between phrasing and DNAR consent, we conducted chi-square tests and logistic regression analyses adjusting for age and sex.

The three studies were designed to investigate different forms of selective phrasing. Study 1 examined whether mentioning potential harms or benefits of cardiopulmonary resuscitation affects agreement with DNAR. Study 2 tested whether the order in which harms and benefits are presented influences decisions. Study 3 assessed whether describing DNAR as a form of “natural” end-of-life care increases acceptance.

The study protocol was approved by the Ethics Committee of Hokkaido University. All procedures adhered to the Declaration of Helsinki and its subsequent amendments.

### Study 1

#### Participants

We recruited 200 Japanese participants from Lancers (https://www.lancers.jp/), the largest crowdsourcing platform in Japan. Prior to recruitment, an a priori power analysis using G*Power [8] was conducted to determine the required sample size for a chi-square test, assuming a medium effect size (Cramér’s *V* = 0.3), an alpha level of 0.05, and a desired power of 0.80. The analysis indicated a minimum sample size of N = 88. Based on this, we aimed to recruit as many participants as our budget permitted.

Participants indicated whether they agreed with a DNAR order in a hypothetical scenario, followed by comprehension questions about the situation. The survey was administered via the Survey Monkey platform. All materials were presented in Japanese and completed on participants’ own digital devices. Participants were randomly assigned to one of two conditions: the harm condition or the benefit condition. On average, it took 2.26 min to complete the study, and participants received ¥88 (equivalent to ¥2,336/hour).

A total of 33 participants were excluded: two did not complete the survey, eight completed it within one minute, and 23 failed at least one of the two comprehension checks. The final sample consisted of 167 adults (106 men, 63 women; *M*_age_ = 44.88, *SD*_age_ = 10.08). The number of participants in each condition was as follows: benefit = 95, harm = 72.

#### Materials and methods

Participants were randomly assigned to either the benefit condition or the harm condition. In the benefit condition, participants were asked to imagine the following situation, which described life-saving measures as beneficial for the patient. All materials were originally presented in Japanese.

You live with your elderly mother. One day, your mother suddenly fell ill and you called an ambulance because you felt something was wrong with her. She was taken to the emergency room and admitted to the hospital. A few days later, while she was in a coma, her doctor told you the following: “We would like to discuss your mother's future medical care. I am very sorry to have to tell you this, but considering her age, it is unlikely that her condition will improve with further treatment. Also, given her current condition, her breathing and heart may stop in the near future. Although it may be difficult to decide immediately because of the suddenness of the situation, if breathing or heart stops in the future, you may choose not to perform life-saving measures such as intubation or CPR. Of course, life-saving measures can be performed if the family wishes. Choosing not to use life-sustaining measures does not mean that we will stop any treatment for your mother. **However, if you do perform life-saving measures, such as intubation with a ventilator and CPR, you may be able to prolong your mother's life slightly.** If her condition worsens in the future and life-saving measures become necessary, would you want them?”.

In contrast, in the harm condition, the bolded portion was replaced with the following:

**However, given her current medical condition, life-saving measures, such as intubation with a ventilator and CPR may cause her to suffer even more.**

After reading the scenario, participants in both conditions were asked to indicate their preferred course of action in the given situation by selecting one of the following options:

If you were faced with the above situation, would you want life-saving measures to be taken for your mother?

I want/I do not want

In both conditions, participants also answered two comprehension questions to assess their understanding of the scenario. These questions were presented in random order:

What do you think would happen to the mother if she did not intubate the ventilator or perform CPR when her breathing and heart stopped?

1. I think there is enough chance that she will survive.
2. I think she would most likely die.

What do you think would happen to the mother after that, even if life-saving measures were actually taken?

1. I think she will recover and be discharged from the hospital.
2. I don't think she will live long.

Participants who chose option 1 for either question were excluded from the final analysis for failing to demonstrate sufficient understanding of the scenario.

### Study 2

In light of the findings from Study 1, we conducted a follow-up investigation to determine whether the observed effects would persist when the information was standardized across conditions. In Study 2, we prepared two descriptions that included both harm- and benefit-related phrasing, differing only in the order in which these two elements were presented.

#### Participants

As in Study 1, we recruited 400 participants through Lancers and conducted the experiment on SurveyMonkey. Prior to recruitment, an a priori power analysis using G*Power [8] was conducted for a chi-square test, which indicated that a sample size of 88 was required to detect a medium effect size (Cramér’s *V* = 0.3) with α = 0.05 and power = 0.80. Based on this, we aimed to recruit as many participants as our budget permitted. On average, participants took 2.48 min to complete the study and received ¥55 (¥1,330/hour).

A total of 78 participants were excluded: 23 completed the survey within one minute, and 55 failed at least one of the two comprehension checks (the same as in Study 1). The final sample consisted of 332 adults (211 men, 121 women; *M*_age_ = 43.96, *SD*_age_ = 9.86). The number of participants in each condition was as follows: benefit = 164, harm = 168.

#### Materials and methods

Participants were randomly assigned to either the benefit or harm condition. In Study 2, the two conditions were created by modifying the sequence in which the key phrases were presented. In both conditions, participants were shown a scenario similar to that used in Study 1. In the benefit condition, the bolded portion of the original scenario was replaced with the following phrase:

Given her current medical condition, life-saving measures, such as intubation with a ventilator and CPR may cause her to suffer even more; **however, if you do perform life-saving measures, such as intubation with a ventilator and CPR, you may be able to prolong your mother's life slightly.**

In contrast, in the harm condition, the same portion was replaced with the following:

If you do perform life-saving measures, such as intubation with a ventilator and CPR, you may be able to prolong your mother's life slightly; **however, given her current medical condition, life-saving measures, such as intubation with a ventilator and CPR may cause her to suffer even more.**

After reading the scenario, participants responded to the same set of questions used in Study 1.

### Study 3

DNAR is often described as a form of “natural end-of-life care” (自然な看取り) in Japan. The concept of “natural end-of-life care” is considered distinctive to Japan. Indeed, a Google search for “自然な看取り” yields more than 4,000 results, and this expression even appears in academic papers written in Japanese and indexed on Google Scholar (65 hits as of June 2026). In contrast, the number of webpages containing the term “natural end-of-life care” in English is quite small (19 hits as of June 2026). Given the several potential concerns related to translating this concept (e.g., there may be alternative English translations that better capture the underlying notion), it would be premature to conclude that this phrasing is unique to Japanese culture. Nevertheless, its prevalence in the Japanese context suggests that “natural end-of-life care” carries positive connotations for many Japanese people, potentially making DNAR orders more acceptable to them. Following Studies 1 and 2, Study 3 examined whether the use of this expression affects people’s attitudes toward DNAR.

#### Participants

As in Study 1, we recruited 200 participants through Lancers and conducted the experiment on SurveyMonkey. Prior to recruitment, an a priori power analysis using G*Power [8] indicated a minimum sample size of N = 88, assuming a medium effect size (Cramér’s *V* = 0.3), an alpha level of 0.05, and power = 0.80. Based on this, we aimed to recruit as many participants as our budget permitted. On average, it took participants 2.83 min to complete the study, and they received ¥88 (¥1,865/hour).

A total of 43 participants were excluded: eight did not complete the survey, nine completed it within one minute, and 26 failed at least one of the two comprehension checks (the same as in Study 1). The final sample consisted of 157 adults (99 men, 58 women; *M*_age_ = 46.05, *SD*_age_ = 8.98). The number of participants in each condition was as follows: natural condition = 82, control condition = 75.

#### Materials and methods

Participants were randomly assigned to one of two conditions. In both conditions, they were presented with a scenario similar to that used in Study 1. In the natural condition, where consent to DNAR was described as a means of allowing a natural end of life, the bolded portion of the original scenario was replaced with the following phrase:

Given her current medical condition, life-saving measures, such as intubation with a ventilator and CPR may cause her to suffer even more; **however, I believe that your mother can be cared for in a natural way if you do not perform life-saving measures, such as intubation with a ventilator and CPR.**

In contrast, in the control condition, consent to DNAR was described as a means of slightly prolonging life:

Given her current medical condition, I believe that your mother can be cared for in a natural way if you do not perform such life-saving measures, such as intubation with a ventilator and CPR; **however, if you do perform life-saving measures, such as intubation with a ventilator and CPR, you may be able to prolong your mother's life slightly.**

The modification in the order of two phrases between conditions resulted in the creation of two distinct descriptions of DNAR. One description emphasized DNAR as a form of natural end-of-life care, whereas the other highlighted that life-saving measures remained available. As in Study 2, the informational content was identical across the two conditions.

After reading each scenario, participants answered the same set of questions as in Study 1, following the same procedure.

## Results

### Study 1

**Fig. 1:**
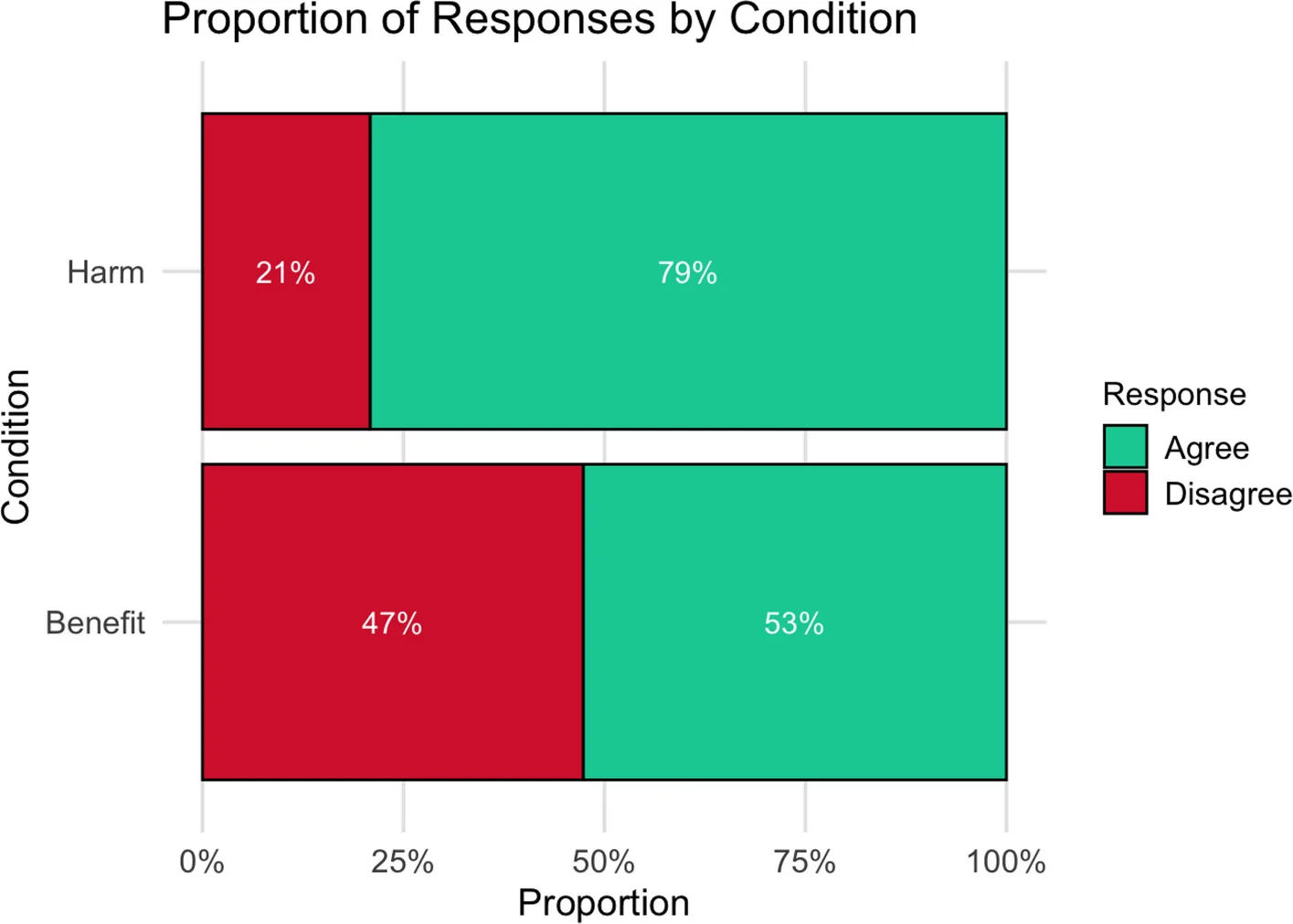
Proportion of agreement and disagreement with DNAR in Study 1 (benefit vs harm condition)

In the benefit condition, 52.63% of respondents agreed with the DNAR recommendation, whereas in the harm condition, as many as 79.26% agreed. The discrepancy in agreement rates was statistically significant (*χ*^2^ = 12.528, *p* < 0.001, adjusted *OR* = 3.42, 95% *CI* [1.73, 7.03], Cramer’s *V* = 0.27). In logistic regression adjusting for age and sex, the harm wording remained associated with higher consent (Condition: adjusted *OR* = 4.30, 95% *CI* [2.09, 9.31], *p* = 0.001, Sex (0:female, 1:male): adjusted *OR* = 0.51, 95% *CI* [0.24, 1.05], *p* = 0.071, age: adjusted *OR* = 1.06, 95% *CI* [1.02, 1.10], *p* = 0.004) (Fig. 1).

### Study 2

**Fig. 2:**
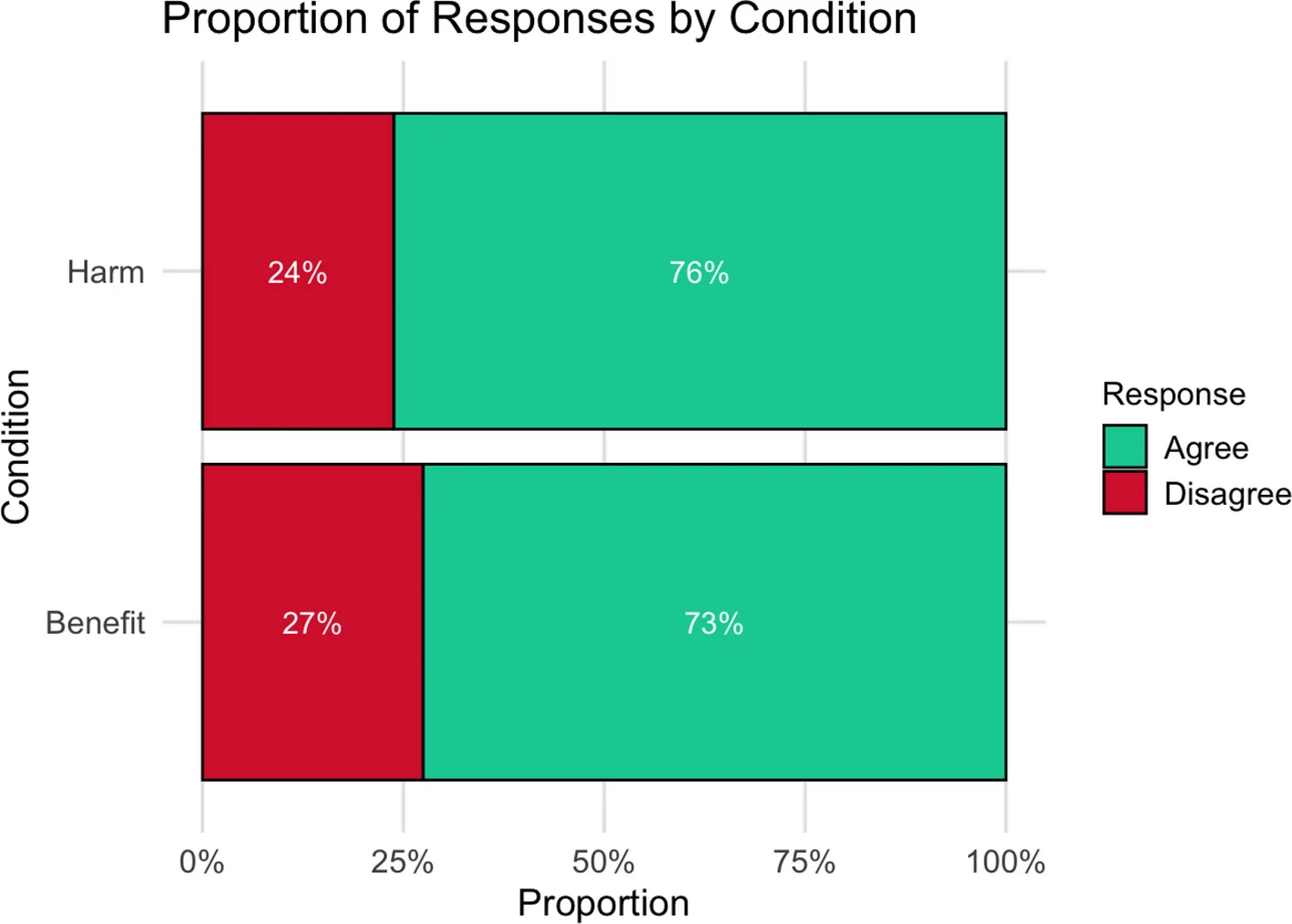
Proportion of agreement and disagreement with DNAR in Study 2 (benefit first vs harm first condition)

The results showed that 76.19% of participants in the harm condition and 72.56% in the benefit condition agreed with the DNAR recommendation. This difference in agreement rates was not statistically significant (*χ*^2^ = 0.574, *p* = 0.449, Cramer’s *V* = 0.04). In logistic regression controlling for age and sex, the condition was not a significant association with consent rate (Condition: adjusted *OR* = 1.18, 95% *CI* [0.71, 1.94], *p* = 0.523, Sex (0:female, 1:male): adjusted *OR* = 0.62, 95% *CI* [0.36, 1.06], *p* = 0.087, age: adjusted *OR* = 0.99, 95% *CI* [0.96, 1.01], *p* = 0.405). Although the difference in condition is not statistically associated with agreement rate, it is noteworthy that in both conditions, the agreement rate was higher than that observed in the benefit condition of Study 1. This suggests that referring to the patient’s potential suffering as a consequence of resuscitation has a substantial impact on agreement with DNAR, irrespective of references to the possibility of recovery (Fig. 2).

### Study 3

**Fig. 3:**
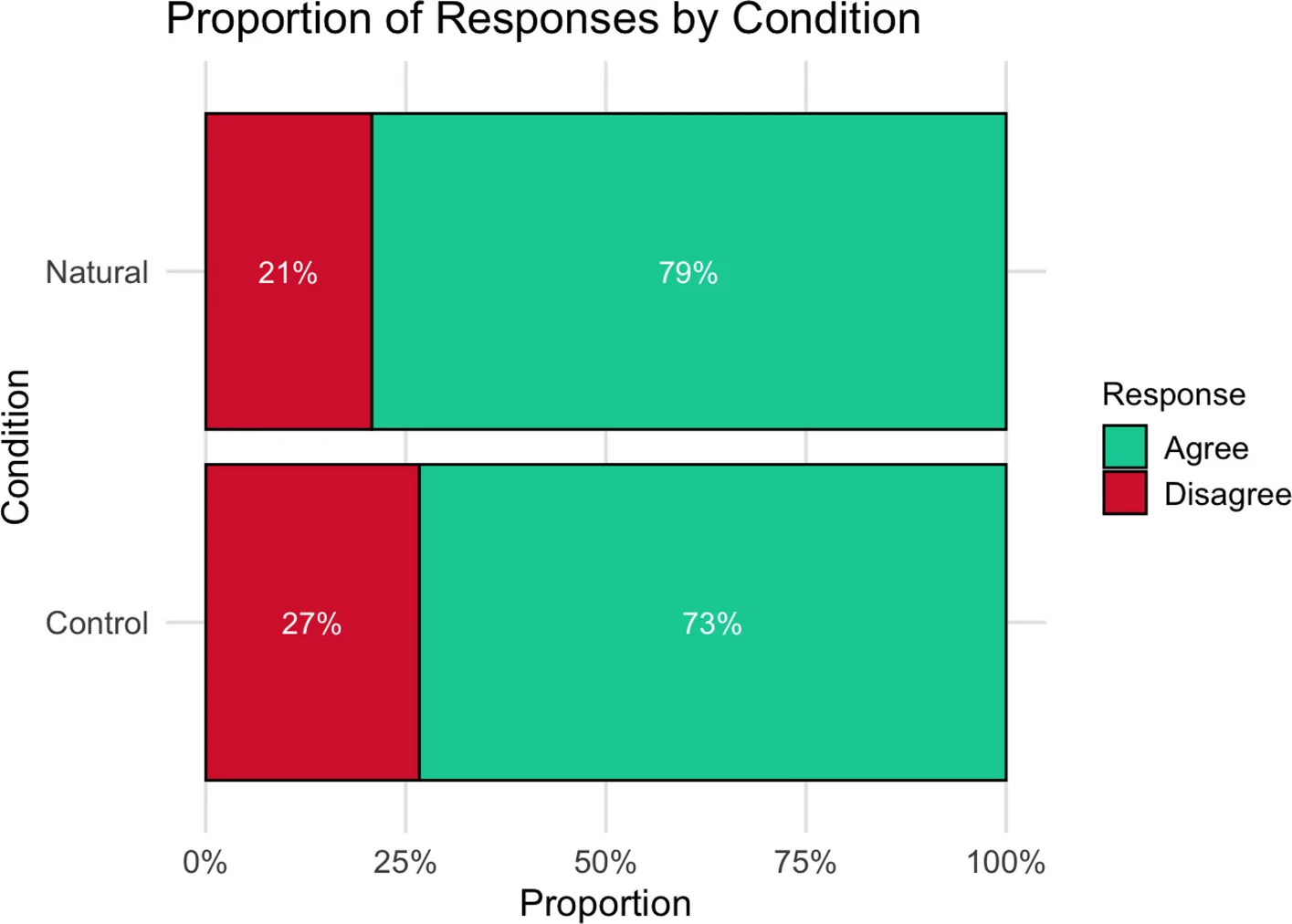
Proportion of agreement and disagreement with DNAR in Study 3 (natural vs control condition)

In both conditions, the proportion of agreement with DNAR was higher than that observed in the benefit condition of Study 1. The data also showed that the proportion of refusals to perform resuscitation was marginally higher in the natural condition, where DNAR was described as a form of “natural end-of-life care.” However, this difference was not statistically significant (*χ*^2^ = 0.766, *p* = 0.382, Cramer’s *V* = 0.07). In logistic regression controlling for age and sex, the condition was not a significant association with consent rate (Condition: adjusted *OR* = 1.40, 95% *CI* [0.64, 3.10], *p* = 0.400, Sex (0:female, 1:male): adjusted *OR* = 0.24, 95% *CI* [0.09, 0.61], *p* = 0.004, age: adjusted *OR* = 1.07, 95% CI [1.02, 1.13], *p* = 0.006) (Fig. 3).

## General discussion

### Empirical contributions

Across three studies, we found that even subtle differences in physicians’ phrasing meaningfully shape people’s attitudes toward DNAR. The findings from Studies 1 and 2 suggest that referencing the potential for a patient to experience harm as a result of resuscitation is associated with increased agreement with DNAR orders. Study 2 showed that merely changing the order of presenting risks and benefits did not significantly alter agreement rates; however, both versions elicited consistently higher^1^ acceptance (harm: 76%, benefit: 73%) compared to the benefit condition in Study 1 (53%). In short, Studies 1 and 2 suggest that mentioning potential harm, regardless of its order, nudges agreement with DNAR.

Furthermore, Study 3 found that using the term “natural” increased DNAR acceptance. Even without mentioning the potential harm caused by CPR, both conditions in Study 3 elicited higher acceptance (natural: 79%, control: 73%) than the benefit condition in Study 1 (53%). This suggests that associating DNAR with “naturalness” can increase agreement even when no explicit reference to CPR-related harm is made. Taken together, these results demonstrate that physicians’ selective phrasing can steer surrogates’ decisions in end-of-life contexts without overtly constraining their freedom of choice.

### Limitations

Several limitations of this research should be acknowledged. First and foremost, it remains unclear whether findings from vignette-style online experiments can be directly generalized to real-life medical situations. Two possibilities merit consideration. On the one hand, the effects observed here may be weaker in actual end-of-life settings, where family members tend to engage in more careful and deliberative reflection. On the other hand, real-world interactions involve numerous additional factors, such as tone of voice, facial expression, and body posture, that may further shape patients’ or their surrogates’ decision-making processes. It is possible that the influence of selective phrasing could be even greater in actual clinical settings than in experimental contexts.

Second, our studies involved only Japanese participants, which may limit the generalizability of the findings. Cross-cultural research is essential for a fuller understanding of these effects. As noted in Study 3, the association between “naturalness” and end-of-life care appears to be culturally specific to Japan. Thus, it is unlikely that a direct translation of “natural end-of-life care” would yield comparable effects in other linguistic or cultural contexts. Future research should examine whether culturally specific expressions in other languages produce similar influences on attitudes toward DNAR.

Third, our experiments focus on situations in which a family member must decide whether to consent to a DNAR order on behalf of a patient. We adopted this design because, in many real-world situations where DNAR decisions become clinically relevant, patients have already lost the capacity to communicate their wishes, leaving such decisions to surrogate decision-makers. Thus, the present findings speak most directly to surrogate decision-making. At the same time, surrogate decision-making is not identical to patients’ own treatment decisions. While the two contexts may share some features, selective phrasing may influence them in different ways. The present findings should therefore not be assumed to apply automatically to patients’ self-regarding choices. Further research is needed to determine whether similar effects emerge when patients make decisions about their own care.

Fourth, the mean age of participants in our experiments was in the mid-40 s. In Studies 1 and 3, the logistic regression analyses revealed significant age effects, with older participants being more likely to agree with DNAR orders. These findings raise the possibility that older individuals may make different judgments from younger individuals, perhaps because they are more likely to have experienced the illness, death, or care of family members and relatives. However, the pattern of results regarding the experimental conditions remained unchanged when age effects were taken into account: the effects of the experimental manipulations were non-significant in both the main analyses (chi-square tests) and the logistic regression models. Further research (e.g., studies with larger samples of older adults, including those aged 60–80 and those over 80) is needed to clarify the relationship between age and attitudes toward DNAR decisions, as well as the extent to which age influences susceptibility to selective phrasing.

### Ethical concern and analysis

As we noted at the beginning of this paper, the moral permissibility of narrow nudges in medical contexts remains contentious. Thus, even if the broad nudges defined in this paper, including selective phrasing, constitute an appropriate expansion of the original concept of nudging, it remains questionable whether the use of such broad nudges is morally permissible. Again, settling the general moral permissibility of nudging is beyond the scope of this paper. Nevertheless, it is important to note several ethical concerns distinctive to selective phrasing.

An important ethical constraint on selective phrasing arises from the disclosure requirements of informed consent. Valid consent requires that a sufficient amount of relevant information be presented to patients. According to Millum and Bromwich [3, 12], the primary purpose of disclosure is to prevent “illegitimate control” over decision-making. Consent is undermined when practitioners withhold or misrepresent relevant information—what they call *fraudulent disclosure*. To avoid such illegitimate influence, practitioners must disclose information they know, or have good reason to believe, is relevant to patients’ decisions and that patients could reasonably expect to be told.

Our findings raise the question of how these requirements apply to DNAR orders. Even when DNAR is expected to serve the patient’s best interests, excessive selectivity—whether by omitting relevant information or by emphasizing certain aspects—may approach fraudulent disclosure. For example, our experiments indicate that mentioning the potential harms of CPR increases consent to DNAR. Yet, following Millum and Bromwich’s framework, physicians may also be required to mention the potential benefits of CPR, since both types of information could influence the decision-making. Although Study 2 shows that providing information about potential benefits has only a limited marginal effect, intentionally omitting the fact that the probability of successful CPR is not zero could constitute a wrongful omission and thereby undermine the validity of informed consent.

Selective phrasing can also influence decisions through emphasis rather than omission. Our findings show that describing DNAR as “natural end-of-life care” increases agreement with DNAR, suggesting that highlighting certain aspects or adding specific information meaningfully shapes surrogates’ decision-making. However, excessive emphasis or the addition of misleading information may distort decision-makers’ understanding of their medical situation. For instance, if a physician were to say that “agreeing to DNAR guarantees a painless death,” this would be an inaccurate and misleading characterization.

Both omission and emphasis raise concerns about whether selective phrasing may constitute a form of fraudulent disclosure. These considerations lead to a broader question: to what extent should physicians be permitted to be selective in their explanations during informed consent? While the present study does not settle this question, it is useful to outline some promising criteria.

### Practical recommendations

First, selective phrasing—especially omission of relevant information—is permissible only when including the information would be likely to mislead decision-makers or distort their understanding of the medical facts. For instance, if decision-makers tend to significantly overestimate the likelihood of successful CPR, omitting explicit mention of the small chance of survival may be ethically permissible. Bromwich and Millum [3, 12] emphasize that disclosed information must be understandable for recipients. If mentioning rare CPR success is likely to create misunderstanding, refraining from doing so could be consistent with appropriate disclosure. In fact, some studies suggest that the success rates of CPR depicted in medical dramas are unrealistically high, potentially distorting public understanding of actual CPR outcomes [1, 15]. Such considerations may provide at least a partial justification for physicians not to place undue emphasis on the slight possibility of successful resuscitation.

Second, selective phrasing that emphasizes one aspect of medical information is permissible only if it does not misrepresent the underlying medical situation. Referring to DNAR as “natural end-of-life care” may be permissible when it accurately reflects the patient’s clinical condition and likely outcomes, but not when it implies an unwarranted level of certainty or oversimplifies the medical context. Expressions whose objective validity as representations of the medical situation is questionable should be avoided.

Third, selective phrasing must be used solely in patients’ interests, rather than for reasons of institutional convenience or resource management. Selectivity motivated by staffing limitations or systemic pressures would not meet the ethical demands of informed consent. While physicians should in principle disclose all information relevant to informed decision-making, selective phrasing can be ethically permissible when it promotes both patients’ interests and accurate understanding of their medical situation.

Lastly, this paper does not suggest that narrow nudges are limited to probabilistic framing. Other forms of narrow nudging, such as interventions that exploit opt-in/opt-out defaults, may remain feasible both in DNAR decision-making and in other medical contexts. The practical challenges associated with probabilistic framing in this particular context do not imply the infeasibility of narrow nudges in general. Moreover, DNAR discussions involve considerations that extend beyond probabilistic expectations, including patients’ values, preferences, and advance directives. Accordingly, DNAR discussions are used here only as an illustrative case rather than as a paradigmatic model of clinical communication. The example serves to highlight a specific challenge: communicating the judgement that an intervention is unlikely to benefit a patient may become psychologically and professionally difficult when that judgement is conveyed through explicit probabilistic framing, one of the most frequently discussed forms of nudging in the healthcare literature. The implications of this paper should therefore be assessed on a case-by-case basis. Whether selective phrasing or other forms of nudging are appropriate in a particular medical context depends on the characteristics of the decision at issue as well as the relevant ethical considerations.

## Conclusion

This study investigated the influence of selective phrasing on consent to DNAR. Through a series of online experiments with vignette-style surveys, we discovered that the inclusion of specific phrases emphasising particular aspects of CPR or DNAR can significantly impact people’s decision-making. Our results suggest that certain phrasings enable physicians to implicitly and effectively recommend DNAR within the practical context of end-of-life care. In other words, they can influence surrogates’ decisions without limiting their options or compromising their autonomy. While further ethical analysis is needed regarding when physicians are permitted to be selective in their word choices, our findings suggest that there are promising ways to nudge people via implicit recommendations and selective phrasing in medical contexts beyond the utilization of cognitive biases.

## Supplementary Information

Supplementary Material 1.

## Data Availability

The datasets generated and analyzed during the current study are available from the corresponding author on reasonable request.

## Abbreviations

CPR: Cardiopulmonary Resuscitation
DNAR: Do Not Attempt Resuscitation

## Footnotes

1 One might worry that a substantial number of participants were excluded due to failing the comprehension checks across the studies. To address this concern, we conducted additional analyses using samples that included participants who failed the comprehension checks in Studies 2 and 3. In both studies, the direction of the effect was consistent with the main analyses, but the differences were not statistically significant (Study 2: *χ*^*2*^(1) = 0.27, *p* =.605; Study 3: *χ*^*2*^(1) = 0.52, *p* =.472). In Study 2 (N = 194 in the Benefit condition and N = 193 in the Harm condition), 29% of participants in the Benefit condition and 32% in the Harm condition agreed with DNAR. In Study 3 (N = 92 in the Control condition and N = 101 in the Natural condition), 29% of participants in the Control condition and 25% in the Natural End-of-Life Care condition agreed with DNAR. This suggests that the exclusion criteria did not substantively alter the primary conclusions regarding the selective phrasings.

## References

[1] Bitter CC, Patel N, Hinyard L. Depiction of resuscitation on medical dramas: proposed effect on patient expectations. Cureus. 2021;13(4):e14419. 10.7759/cureus.14419. PMC8112599 33987068

[2] Blumenthal-Barby JS. Between reason and coercion: ethically permissible influence in health care and health policy contexts. Kennedy Inst Ethics J. 2012;22(4):345–66. 23420941

[3] Bromwich D, Millum J. Disclosure and consent to medical research participation. J Moral Philos. 2013;10(4):195–219. 10.1163/17455243-4681027.

[4] Chwang E. Consent’s been framed: when framing effects invalidate consent and how to validate it again. J Appl Philos. 2015;33(3):270–85. 10.1111/japp.12112.

[5] Cohen S. Nudging and informed consent. Am J Bioeth. 2013;13(6):3–11. 10.1080/15265161.2013.781704. 23641835

[6] Director S. Framing effects do not undermine consent. Ethic Theory Moral Pract. 2024;27(2):221–35. 10.1007/s10677-023-10412-1.

[7] Douglas C, Proudfoot E. Nudging and the complicated real life of “informed consent.” Am J Bioeth. 2013;13(6):16–7. 10.1080/15265161.2013.781716. 23641838

[8] Faul F, Erdfelder E, Lang A-G, Buchner A. G*Power 3: a flexible statistical power analysis program for the social, behavioral, and biomedical sciences. Behav Res Methods. 2007;39(2):175–91. 10.3758/bf03193146. 17695343

[9] Holm S, Ploug T. On nudging and informed consent-four key undefended premises. Am J Bioeth. 2013;13(6):31–3. 10.1080/15265161.2013.781717. 23641846

[10] Kiener M. When do nudges undermine voluntary consent? Philos Stud. 2021;178(12):4201–26. 10.1007/s11098-021-01644-x. PMC8536605 34720203

[11] Malloy TR, Wigton RS, Meeske J, Tape TG. The influence of treatment descriptions on advance medical directive decisions. J Am Geriatr Soc. 1992;40(12):1255–60. 10.1111/j.1532-5415.1992.tb03652.x. 1447444

[12] Millum J, Bromwich D. Informed consent: what must be disclosed and what must be understood? Am J Bioeth. 2021;21(5):46–58. 10.1080/15265161.2020.1863511. 33460362

[13] Munoz R, Fox M, Gomez M, Gelfand S. Evidence-Based Nudging: Best Practices in Informed Consent. The American Journal of Bioethics: AJOB. 2015;15(10):43–5. 10.1080/15265161.2015.1074311. 26479102

[14] Parmer WJ. Nudges, nudging, and self-guidance under the influence. Ergo. 2023;9(44):1199–232. 10.3998/ergo.2913.

[15] Portanova J, Irvine K, Yi JY, Enguidanos S. It isn’t like this on TV: Revisiting CPR survival rates depicted on popular TV shows. Resuscitation. 2015;96:148–50. 10.1016/j.resuscitation.2015.08.002. 26296584

[16] Raskoff SZ. Nudges and hard choices. Bioethics. 2022;36(9):948–56. 10.1111/bioe.13091. PMC9826097 36152342

[17] Simkulet W. Nudging, informed consent and bullshit. J Med Ethics. 2018;44(8):536–42. 10.1136/medethics-2017-104480. 29151058

[18] Thaler RH, Sunstein CR. Nudge: improving decisions about health, wealth, and happiness. Yale University Press; 2008.

[19] Tversky A, Kahneman D. The framing of decisions and the psychology of choice. Science. 1981;211(4481):453–8. 10.1126/science.7455683. 7455683

[20] Yeo SL. Framing effects from misleading implicatures: an empirically based case against some purported nudges. J Med Ethics. 2025;51(7):487–92. 10.1136/jme-2024-110015. 39209374

