# Supplementary material for: Influence of selective phrasing on consent to DNAR
Source: BMC Med Ethics. 2026 Jul 14;27:228. doi: 10.1186/s12910-026-01548-7 (PMC13741579; doi:10.1186/s12910-026-01548-7)
Supplement: Supplementary file 1 [file 12910_2026_1548_MOESM1_ESM.docx]

**Experimental Materials**

*All materials were originally presented in Japanese

1. **Consent Form and Demographic questions.**

Across Studies 1–3, all participants took part in the experiment after reading the following consent statement. After confirming the Consent form, participants responded to the statement, “After confirming the above, press the button below to begin answering,” and only those who checked “I agree with the above and will now start my response” were allowed to proceed to the experiment.

Also, in all studies, participants were asked to report their gender and age as demographic information, and to enter their ID on the crowdsourcing platform (Lancers).

**Consent Form**

Thank you very much for taking part in this survey, which is being conducted by the Experimental Philosophy Group at Hokkaido University.

This research project explores your personal views on issues related to medical ethics. You will be asked to respond to a hypothetical scenario involving the care of a family member in hospital. You will be asked to consider the perspectives of the different characters in the scenario and indicate what decision you would make if you were in their position.

All responses will be processed anonymously by computer for statistical analysis and will not be used for any other purpose. Please respond honestly.

This survey is optimised for response via a computer browser. Please use full-screen mode when answering.

―Response notes―

You may feel that some questions are repetitive, but each one is necessary and important.

Do not press your browser's 'Back' button. Pressing the 'Back' button will prevent your answers from being saved correctly. Also, do not press the 'Backspace' key on your keyboard except when correcting text entered into text boxes. Doing so may cause errors.

Once you have finished answering the questions, we will display the code required to receive your reward via crowdsourcing.

**Demographic Questions (Common Across Study1-3)**

- Please tell us your gender.
- Please tell us your age.
- Enter your Lancers ID.

2. **Scenarios and Question: Study1**

Participants were randomly assigned to either of the two conditions. After reading the scenario, participants in both conditions were asked to indicate the decision of action in the given situation by selecting “I want” or “I do not want”. Across all studies, the wording of the scenario varied slightly across studies; however, the questions and response options were the same.

**Benefit Condition**

You live with your elderly mother. One day, your mother suddenly fell ill and you called an ambulance because you felt something was wrong with her. She was taken to the emergency room and admitted to the hospital. A few days later, while she was in a coma, her doctor told you the following: “We would like to discuss your mother's future medical care. I am very sorry to have to tell you this, but considering her age, it is unlikely that her condition will improve with further treatment. Also, given her current condition, her breathing and heart may stop in the near future. Although it may be difficult to decide immediately because of the suddenness of the situation, if breathing or heart stops in the future, you may choose not to perform life-saving measures such as intubation or CPR. Of course, life-saving measures can be performed if the family wishes. Choosing not to use life-sustaining measures does not mean that we will stop any treatment for your mother. **However, if you do perform life-saving measures, such as intubation with a ventilator and CPR, you may be able to prolong your mother's life slightly**. If her condition worsens in the future and life-saving measures become necessary, would you want them?”

Question: If you were faced with the above situation, would you want life-saving measures to be taken for your mother?

**Harm Condition**

You live with your elderly mother. One day, your mother suddenly fell ill and you called an ambulance because you felt something was wrong with her. She was taken to the emergency room and admitted to the hospital. A few days later, while she was in a coma, her doctor told you the following: “We would like to discuss your mother's future medical care. I am very sorry to have to tell you this, but considering her age, it is unlikely that her condition will improve with further treatment. Also, given her current condition, her breathing and heart may stop in the near future. Although it may be difficult to decide immediately because of the suddenness of the situation, if breathing or heart stops in the future, you may choose not to perform life-saving measures such as intubation or CPR. Of course, life-saving measures can be performed if the family wishes. Choosing not to use life-sustaining measures does not mean that we will stop any treatment for your mother. **However, given her current medical condition, life-saving measures, such as intubation with a ventilator and CPR may cause her to suffer even more.** If her condition worsens in the future and life-saving measures become necessary, would you want them?”

Question: If you were faced with the above situation, would you want life-saving measures to be taken for your mother?

3. **Scenarios and Question: Study2**

**Benefit Condition**

You live with your elderly mother. One day, your mother suddenly fell ill and you called an ambulance because you felt something was wrong with her. She was taken to the emergency room and admitted to the hospital. A few days later, while she was in a coma, her doctor told you the following: “We would like to discuss your mother's future medical care. I am very sorry to have to tell you this, but considering her age, it is unlikely that her condition will improve with further treatment. Also, given her current condition, her breathing and heart may stop in the near future. Although it may be difficult to decide immediately because of the suddenness of the situation, if breathing or heart stops in the future, you may choose not to perform life-saving measures such as intubation or CPR. Of course, life-saving measures can be performed if the family wishes. Choosing not to use life-sustaining measures does not mean that we will stop any treatment for your mother. Given her current medical condition, life-saving measures, such as intubation with a ventilator and CPR may cause her to suffer even more; **however, if you do perform life-saving measures, such as intubation with a ventilator and CPR, you may be able to prolong your mother's life slightly.** If her condition worsens in the future and life-saving measures become necessary, would you want them?”

Question: If you were faced with the above situation, would you want life-saving measures to be taken for your mother?

**Harm Condition**

You live with your elderly mother. One day, your mother suddenly fell ill and you called an ambulance because you felt something was wrong with her. She was taken to the emergency room and admitted to the hospital. A few days later, while she was in a coma, her doctor told you the following: “We would like to discuss your mother's future medical care. I am very sorry to have to tell you this, but considering her age, it is unlikely that her condition will improve with further treatment. Also, given her current condition, her breathing and heart may stop in the near future. Although it may be difficult to decide immediately because of the suddenness of the situation, if breathing or heart stops in the future, you may choose not to perform life-saving measures such as intubation or CPR. Of course, life-saving measures can be performed if the family wishes. Choosing not to use life-sustaining measures does not mean that we will stop any treatment for your mother. If you do perform life-saving measures, such as intubation with a ventilator and CPR, you may be able to prolong your mother's life slightly**; however, given her current medical condition, life-saving measures, such as intubation with a ventilator and CPR may cause her to suffer even more**. If her condition worsens in the future and life-saving measures become necessary, would you want them?”

Question: If you were faced with the above situation, would you want life-saving measures to be taken for your mother?

4. **Scenarios and Question: Study3**

**Natural Condition**

You live with your elderly mother. One day, your mother suddenly fell ill and you called an ambulance because you felt something was wrong with her. She was taken to the emergency room and admitted to the hospital. A few days later, while she was in a coma, her doctor told you the following: “We would like to discuss your mother's future medical care. I am very sorry to have to tell you this, but considering her age, it is unlikely that her condition will improve with further treatment. Also, given her current condition, her breathing and heart may stop in the near future. Although it may be difficult to decide immediately because of the suddenness of the situation, if breathing or heart stops in the future, you may choose not to perform life-saving measures such as intubation or CPR. Of course, life-saving measures can be performed if the family wishes. Choosing not to use life-sustaining measures does not mean that we will stop any treatment for your mother. Given her current medical condition, life-saving measures, such as intubation with a ventilator and CPR may cause her to suffer even more; **however, I believe that your mother can be cared for in a natural way if you do not perform life-saving measures, such as intubation with a ventilator and CPR.** If her condition worsens in the future and life-saving measures become necessary, would you want them?”

Question: If you were faced with the above situation, would you want life-saving measures to be taken for your mother?

**Control Condition**

You live with your elderly mother. One day, your mother suddenly fell ill and you called an ambulance because you felt something was wrong with her. She was taken to the emergency room and admitted to the hospital. A few days later, while she was in a coma, her doctor told you the following: “We would like to discuss your mother's future medical care. I am very sorry to have to tell you this, but considering her age, it is unlikely that her condition will improve with further treatment. Also, given her current condition, her breathing and heart may stop in the near future. Although it may be difficult to decide immediately because of the suddenness of the situation, if breathing or heart stops in the future, you may choose not to perform life-saving measures such as intubation or CPR. Of course, life-saving measures can be performed if the family wishes. Choosing not to use life-sustaining measures does not mean that we will stop any treatment for your mother. Given her current medical condition, I believe that your mother can be cared for in a natural way if you do not perform such life-saving measures, such as intubation with a ventilator and CPR; **however, if you do perform life-saving measures, such as intubation with a ventilator and CPR, you may be able to prolong your mother's life slightly.** If her condition worsens in the future and life-saving measures become necessary, would you want them?”

Question: If you were faced with the above situation, would you want life-saving measures to be taken for your mother?

5. **Comprehension Questions (Common across all studies)**

Across all studies, participants answered the following two comprehension questions to assess their understanding of the scenario. These questions were presented in random order.

**Comprehension question 1**

What do you think would happen to the mother if she did not intubate the ventilator or perform CPR when her breathing and heart stopped?

- I think there is enough chance that she will survive.
- I think she would most likely die.

**Comprehension question 2**

What do you think would happen to the mother after that, even if life-saving measures were actually taken?

- I think she will recover and be discharged from the hospital.
- I don’t think she will live long.
